# Nurse‐led group information for patients with breast cancer: Equal to individual information? A comparative study

**DOI:** 10.1002/nop2.643

**Published:** 2020-10-04

**Authors:** Karin Brochstedt Dieperink, Elisabeth Ellegaard, Anja Langkjær Astrup, Henriette Tind Hasse, Caroline Matilde Elnegaard, Jeanette Dupont Jensen

**Affiliations:** ^1^ Department of Oncology Research Unit of Oncology Odense University Hospital Odense Denmark; ^2^ Department of Clinical Research University of Southern Denmark Odense Denmark

**Keywords:** breast cancer, family caregivers, information, nurse‐led, peers, self‐efficacy, support

## Abstract

**Aim:**

To investigate outpatients with breast cancer perception of information before and after changed informational practice.

**Design:**

The design was a comparative study.

**Method:**

Information about breast cancer treatment and chemotherapy toxicity changed from individual to nurse‐led group information. Women with early‐stage breast cancer were eligible. To evaluate individual versus group information, the patients completed a questionnaire at their third cycle of chemotherapy, including *Knowledge* of treatment, *Support* from healthcare professionals or peers and general self‐efficacy *Ability to act* in everyday life. The study is registered in OSF https://osf.io/bh7wg.

**Results:**

In total, 90 participants in two groups were included: (a) individual information (*N* = 44) and (b) group information (*N* = 46). Groups were comparable in age and educational level. Both groups found the information satisfactory, with no significant differences regarding perceived knowledge or support. Five of ten questions in self‐efficacy showed significantly better outcomes in patients receiving group information but with no difference in overall self‐efficacy. Group information was non‐inferior compared with individual information. Patients were satisfied in both groups.

## 1 INTRODUCTION

To treat patients with breast cancer includes comprehensive information and resources to prepare patients for treatment and the risk of adverse effects. New ways of organizing information practices should continue to maintain a high level of patient knowledge.

The need for efficacy in the healthcare system has never been greater. There is a growing demand for healthcare services, partly explained by an increase in the overall mean survival age (Danckert et al., 2018) and the development of new technologies and treatments for previously untreatable diseases. This is positive for the individual but poses a challenge for healthcare providers. The increase in demand increases the cost of healthcare services, which requires finding novel ways of decreasing the use of resources without lowering the standard of services provided.

Although that patients' rights may vary in different countries and in different jurisdictions, healthcare providers are obligated to ensure that patients can make informed decisions regarding their own health or a specific treatment and that these decisions are respected (World Health Organization, 1994). The patient must give informed consent before any treatment commences. Therefore, information provided by healthcare professionals should be given in an understandable and meaningful way to ensure that patients can make informed treatment decisions.

## 2 BACKGROUND

Breast cancer is the most common malignancy in women. Worldwide, 1,671,149 new cases of breast cancer were identified in 2012 (Ghoncheh et al., 2016) and approximately 4,668 women and 36 men are diagnosed annually with breast cancer in Denmark (Danish Health Data Authority, 2015). The increase in incidence and improved survival means more patients require specialized information regarding prognosis, treatment and possible adverse effects. Adverse effects related to breast cancer treatment which patients regard as significant include nausea, fatigue, lack of appetite, loss of hair and irritation of the mucous membranes (Anampa et al., 2015). It is important that the patients know which adverse effects to expect and how to minimize them. Adjuvant treatment for early‐stage breast cancer is increasingly complicated, comprising of chemotherapy (taxanes and anthracyclines), endocrine therapy, antibodies and radiation. Patients may struggle to perceive and understand large amounts of information, about prognosis, treatment and adverse effects due to information overload (Ormel et al., 2020). However, individual information for new patients is time‐consuming and depends on the person giving the information. An alternative is to deliver standardized information to a group of patients who can also benefit from sharing knowledge with peers in the same situation. Further, when giving information, it has proven beneficial to provide an opportunity for family caregivers to participate in hearing the information, as family often provide the main support for the patient, especially in the outpatient clinic (Andersen et al., 2019).

Studies show that patients with cancer benefit significantly from peer contact. A Swedish randomized controlled trial (*N* = 382) reported a statistically significant reduction in anxiety among women newly diagnosed with breast cancer when they had an intervention of attending a 1‐week support group seminar and a 4‐day follow‐up 2 months later. The control group was subjected to unspecified standard follow‐up routines (Björneklett et al., 2012). The Netherland Cancer institute—Antoni Van Leeuwenhoek Hospital implemented group information for patients with breast cancer and family caregivers every 2 weeks with continuous evaluation. The group information resulted in the hospital saving approximately 20 nurse hours per week, and both the patients and the nurses were satisfied. In the Dutch study, there was no report of patients feeling overlooked or unable to ask questions in the group settings (van Ooij Oostrom et al., 2010). In a Danish study, a group of 14 patients with head and neck cancer met once a week during treatment for education on treatment and also peer discussions and learning. In this study, the overall experiences were positive, with patients reporting that they did not feel isolated and some expressed uncertainty as to whether they would have completed the treatment without the support of the group (Iversen, 2010). In England, a study investigated whether group consultations had a positive effect on men with prostate cancer. A total of 331 patients were included and randomized into an intervention group (*N* = 166) and a control group (*N* = 165). The study found a significant reduction (*p* = .009) of depression in the group that had attended the group consultations and a significant (*p* = .045) reduction in concerns about treatment procedures (Schofield et al., 2016). However, none of the above‐mentioned studies reported on how the group information was perceived by the patients in terms of level of knowledge or if the group interaction made the patients feel more supported and able to act appropriately towards unexpected issues. Hence, there is a need for further knowledge regarding patients with breast cancer ability to act accordingly when information is provided at a group level and whether group information and interaction facilitate positive feelings of empowerment in the individual patient.

This study aimed to investigate patients with breast cancer perceived knowledge, support from healthcare professionals and peers and the patients' ability to act regarding breast cancer treatment before and after the change in informational practice from individual information to group information with family caregivers and peers.

## 3 METHODS

### 3.1 Design

The study included a change in informational practice as intervention. We used a prospective comparative experimental design (Thiese, 2014) that reports on Knowledge, Perceived support and Ability to act after two groups being provided with either (a) individual information or (b) group information. The guidance by Thiese is followed as a reporting guideline (Thiese, 2014).

### 3.2 Method

#### 3.2.1 Data collection

The study was executed at the outpatient clinic at Department of Oncology, Odense University Hospital. The (a) group with individual information was included in the period 1/1 2016 – 31/3 2016, and the (b) group having group information was included from 1/4–30/6 2016.

#### 3.2.2 Participants

To include patients with no prior knowledge of breast cancer or chemo toxicity, we had the following criteria:

- Inclusion criteria: patients radically operated for early‐stage breast cancer and subsequently referred to adjuvant chemotherapy with curative intent with epirubicin and cyclophosphamide three times at 3‐week intervals followed by either Docetaxel three times at 3‐week intervals or paclitaxel nine times at 1‐week intervals.
- Exclusion criteria: Patients referred to neoadjuvant treatment, as they were treated immediately, hence could not wait for group information. Patients with metastatic disease treatment; patients unable to wait for up to 1 week for information; patients who did not speak Danish; and patients considered by the physician to be unsuitable to participate in a group context, for example due to fragile mental state or cognitive deficit or communicative disabilities such as deafness. In all these cases, an individual interview with a nurse was offered.

#### 3.2.3 Change of informational practice

Information about the treatment plan was provided by a physician and continued to be individual to all patients, but the informational practice about treatment practicalities and the possible adverse effects of chemotherapy changed from individual information to nurse‐led group information before start of treatment.

The group information included a group of 3–6 new patients with breast cancer and 3–6 family caregivers. The group information lasted 1 hr once a week with a new group of patients. The group information was executed at the hospital in a non‐clinical meeting room and was led by two nurses with extensive experience in breast cancer management and in chemotherapy toxicity. The information included topics about disease‐specific information for breast cancer, possible acute physical adverse effects, psychological reactions, possible late adverse effects, guidance on self‐care and family care and information on the follow‐up programme after breast cancer (Danish Health Authority, 2015). Furthermore, it included information on practicalities like dispensing nausea medicine, completing wig request forms and information on adverse effects registration. The topics were presented by a skilled breast cancer nurse using PowerPoint and included the possibility of questions and peer discussions.

#### 3.2.4 Evaluation measures

**FIGURE 1:**
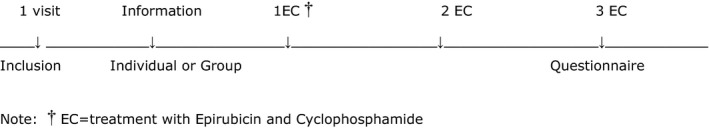
Treatment schedule and data collection

The patients filled out a questionnaire at their third cycle of chemotherapy with epirubicin and cyclophosphamide (Figure 1). The time point of evaluation was because this was just before the patients had to have additional information about the next chemotherapy paclitaxel.

Evaluation measures included a questionnaire designed for the purpose with demographics: age, living situation and educational level. Further, the questionnaire included 26 items containing elements of needed and perceived *Knowledge* of breast cancer, perceived *Support* from the healthcare professionals or peers in different situations and the Danish version of the ten‐item validated general self‐efficacy scale (GSE) questionnaire to investigate the *Ability to act* towards problems in everyday life. The GSE covers a broad range of the sense of personal competence to deal effectively with stressful situations (Schwarzer & Jerusalem, 1995). The scale has been shown to have high validity and reliability in various populations across contexts and cultures (Luszczynska et al., 2005).

### 3.3 Analysis

A 6‐month study period representing 50% of the 1‐year population of the department was chosen, taking into account a non‐participation/dropout rate of 20% as expected in interventional studies (Thiese, 2014) to enable an inclusion rate of approximately 30% of the 1‐year population at the department.

Socio‐demographic and patient‐reported outcomes are presented using means for continuous variables, standard deviations (*SD*) and frequencies for categorical variables.

The general self‐efficacy scale (GSE) comprises ten items, including: “I can always manage to solve difficult problems if I try hard enough.” For each statement, the patients were asked to indicate the extent to which it characterized them as a person. The raw scores of the personal items range from 1 (not at all true)–4 (exactly true). The sum score of the ten items are reported. There is no cut‐off score in the GSE. Higher sum scores represent a higher level of self‐efficacy. The use of resources was calculated in nursing hours per week. Two‐sided *p*‐values were reported, and *p* < .05 were considered statistically significant. Statistics were calculated with STATA 11.

### 3.4 Ethics

Informed consent was obtained from all participants included in the study. Information about the study was given verbally and in writing. The participants were free to withdraw from the study at any time in accordance with the 1964 Helsinki declaration and its later amendments (World Medical Association, 2018). The study was registered at the Danish Data Protection Agency with no. 16/9099. All patients had the opportunity to raise and discuss individual issues with healthcare professionals if requested. Data are secured in a safe Sharepoint site and available by contacting first author.

## 4 RESULTS

### 4.1 Participants

A total of 90 women with breast cancer with a mean age of 57 were included, and 3 patients were excluded according to fragile mental state or cognitive deficit as noted in the exclusion criteria. Five women were informed about the study but did not want to participate in the study due to reasons like fatigue or the situation felt too overwhelming. Further, some women (*N* = 23) were not invited to participate in the study during the inclusion period due to the nurses' workload or omission.

Individual information was provided to 44 women, and their caregivers and group information were provided to 46 women and their caregivers. The groups were comparable in age, living situations and educational level (Table 1). We did not have information about comorbidity status, as this is not systematically documented in the medical files.

**TABLE 1:** Socio‐demographic data of 90 female breast cancer patients before (individual information) and after (group information) change of information practice in relation to treatment

*N* = 90	Individual information (*n* = 44)	Group information (*n* = 46)	*p*‐value
Age, mean (*SD*)	56.2 (9.8)	57.0 (10.3)	.72^a^
Living situation			.71^b^
Living alone, *n* (%)	9 (20.5%)	8 (17.4%)	
Living with partner/spouse, *n* (%)	33 (75.0%)	34 (73.9%)	
Other (living with children), *n* (%)	2 (4.6%)	4 (8.7%)	
Education			.87^b^
Less than 10 years, *n* (%)	9 (20.5%)	9 (19.6%)	
Youth (high school or similar), *n* (%)	13 (29.6%)	12 (26.1%)	
Medium (profession), *n* (%)	19 (43.2%)	21 (45.7%)	
Higher (university), *n* (%)	2 (4.6%)	4 (8.7%)	
Missing	1 (2.27%)	0 (0.0%)	

^a^ *t* test. *p*‐values are two‐sided and <.05 were considered statistically significant.

^b^ Chi‐squared test.

### 4.2 Knowledge

No significant differences between groups were observed regarding knowledge of breast cancer treatment (Table 2) or support from healthcare professionals or peers (Table 3). Patients in both groups had high expectations of the level of knowledge they should have. The perceived knowledge after the information was lower than knowledge needed. In general, the patients were satisfied and assessed the oral information as either really good or good, the individual information group with 95.5% and the group information with 97.1%. Most patients in both groups wanted combined oral and written information, individual information (*N* = 34; 77.3%) and group information (*N* = 41; 89.1%). Two patients in the group information assessed the possibility of asking questions to the staff as bad and equally in both groups 11%–13% of the patients reported that they lacked specific information (Table 2). The topics of this perceived specific missing information were primarily noted as: information about the prognosis, information about diet or information about what they could do themselves.

**TABLE 2:** Knowledge of breast cancer treatment

*N* = 90	Individual information (*n* = 44)	Group information (*n* = 46)	*p*‐value
Knowledge level (patient needed), mean (*SD*)*	9.4 (1.1)	9.3 (1.2)	.89^a^
Knowledge level (perceived), mean (*SD*)*	8.0 (1.8)	8.5 (1.2)	.26^a^
Missing, *n*		1	
Assessment of oral information from the clinic			.67^a^
Really good, *n* (%)	22 (50.0%)	27 (58.7%)	
Good, *n* (%)	20 (45.5%)	18 (39.1%)	
Bad, *n* (%)	1 (2.3%)	1 (2.3%)	
Really bad, *n* (%)	1 (2.3%)	0 (0.0%)	
Preferred way to receive information			.38^a^
Written, *n* (%)	3 (6.8%)	1 (2.2%)	
Oral, *n* (%)	6 (13.6%)	4 (8.7%)	
Both, *n* (%)	34 (77.3%)	41 (89.1%)	
Do not want information, *n* (%)	0 (0.0%)	0 (0.0%)	
Missing, *n* (%)	1 (2.3%)	0 (0.0%)	
Assessment of possibility of asking personnel questions			.38^a^
Really good, *n* (%)	31 (70.5%)	32 (69.6%)	
Good, *n* (%)	12 (27.3%)	12 (26.1%)	
Bad, *n* (%)	0 (0.0%)	2 (4.4%)	
Really bad, *n* (%)	0 (0.0%)	0 (0.0%)	
Missing, *n* (%)	1 (2.3%)	0 (0.0%)	
Special kind of missing information			.91^a^
Yes, *n* (%)	5 (11.4%)	6 (13.0%)	
No, *n* (%)	29 (65.9%)	29 (63.0%)	
Don't know, *n* (%)	9 (20.5%)	11 (23.9%)	
Missing, *n* (%)	1 (2.3%)	0 (0.0%)	

^a^ Chi‐squared test.

* Variable defined as a level of knowledge on a scale from 0–10.

**TABLE 3:** Support from the staff in the hospital department

*N* = 90	Individual information (*n* = 44)	Group information (*n* = 46)	*p*‐value
Support in handling physical problems (like nausea)			.34^a^
Really good, *n* (%)	35 (79.6%)	32 (69.6%)	
Good, *n* (%)	6 (13.6%)	12 (26.1%)	
Bad, *n* (%)	0 (0.0%)	1 (2.2%)	
Really bad, *n* (%)	0 (0.0%)	0 (0.0%)	
Not relevant, *n* (%)	2 (4.6%)	1 (2.2%)	
Missing, *n* (%)	1 (2.3%)	0 (0.0%)	
Support in handling family problems			.63^a^
Really good, *n* (%)	10 (22.7%)	8 (17.4%)	
Good, *n* (%)	6 (13.6%)	9 (19.6%)	
Bad, *n* (%)	0 (0.0%)	1 (2.2%)	
Really bad, *n* (%)	0 (0.0%)	0 (0.0%)	
Not relevant, *n* (%)	27 (61.4%)	28 (60.9%)	
Missing, *n* (%)	1 (2.3%)	0 (0.0%)	
Support in psychological problems			.45^a^
Really good, *n* (%)	16 (36.4%)	12 (26.1%)	
Good, *n* (%)	12 (27.3%)	18 (39.1%)	
Bad, *n* (%)	0 (0.0%)	1 (2.2%)	
Really bad, *n* (%)	0 (0.0%)	0 (0.0%)	
Not relevant, *n* (%)	14 (31.8%)	15 (32.6%)	
Missing, *n* (%)	2 (4.6%)	0 (0.0%)	
Support in handling sexual problems			.53^a^
Really good, *n* (%)	5 (11.4%)	3 (6.5%)	
Good, *n* (%)	4 (9.1%)	7 (15.2%)	
Bad, *n* (%)	0 (0.0%)	0 (0.0%)	
Really bad, *n* (%)	0 (0.0%)	0 (0.0%)	
Not relevant, *n* (%)	34 (77.3%)	36 (78.3%)	
Missing, *n* (%)	1 (2.3%)	0 (0.0%)	
Support in handling work‐related problems			.83^a^
Really good, *n* (%)	8 (18.2%)	7 (15.2%)	
Good, *n* (%)	8 (18.2%)	6 (13.0%)	
Bad, *n* (%)	1 (2.3%)	1 (2.2%)	
Really bad, *n* (%)	0 (0.0%)	0 (0.0%)	
Not relevant, *n* (%)	26 (59.1%)	32 (69.6%)	
Missing, *n* (%)	1 (2.3%)	0 (0.0%)	
Need for extra medical contact in last 2 weeks			.46^a^
No, *n* (%)	26 (59.1%)	35 (76.1%)	
Yes, *n* (%)	4 (9.1%)	3 (6.5%)	
Once, *n* (%)	11 (25.0%)	7 (15.2%)	
Twice or more, *n* (%)	2 (4.6%)	1 (2.2%)	
Missing, *n* (%)	1 (2.3%)	0 (0.0%)	
Assessment of having peer support			.57^a^
Really good, *n* (%)	8 (18.2%)	11 (23.9%)	
Good, *n* (%)	13 (29.6%)	13 (28.3%)	
Bad, *n* (%)	1 (2.3%)	4 (8.7%)	
Really bad, *n* (%)	0 (0.0%)	0 (0.0%)	
Not relevant, *n* (%)	18 (40.9%)	17 (37.0%)	
Missing, *n* (%)	4 (9.1%)	1 (2.2%)	

^a^ Chi‐squared test.

### 4.3 Support

The patients assessed support in handling physical problems as most relevant and best perceived, either really good or good in the individual information at 93.2% and in the group information at 95.7% (Table 3). Peer support was assessed as really good or good in the individual information at 47.8% and a small increase in the group information at 52.2%. A relatively large number of patients did not find it relevant with support in handling family problems, individual information 61.4% and group information 60.9%. The same was observed in regard to support in handling sexual problems, which were measured not relevant in the individual information at 77.3% and in the group information at 78.3%.

### 4.4 Self‐efficacy

Two questionnaires had missing data in the GSE scale and were not included in the GSE calculation. In five out of ten questions in the self‐efficacy scale, statistically significant better outcomes were found in patients who had received group information, but the sum score of general self‐efficacy was not significantly different between groups (Table 4).

**TABLE 4:** Assessment of handling problems in everyday life—GSE self‐efficacy

*N* = 90	Individual information (*n* = 44)	Group information (*n* = 46)	*p*‐value
Can solve difficult problems if I try hard enough			.54^a^
Not true, *n* (%)	0 (0.0%)	0 (0.0%)	
True a few times, *n* (%)	3 (6.8%)	1 (2.2%)	
True more times, *n* (%)	21 (47.7%)	23 (50.0%)	
Exactly true, *n* (%)	18 (40.9%)	21 (45.7%)	
Missing, *n* (%)	2 (4.6%)	1 (2.2%)	
I find a way to get what I want			.70^a^
Not true, *n* (%)	4 (9.1%)	3 (6.5%)	
True a few times, *n* (%)	9 (20.5%)	6 (13.0%)	
True more times, *n* (%)	15 (34.1%)	20 (43.5%)	
Exactly true, *n* (%)	14 (31.8%)	15 (32.6%)	
Missing, *n* (%)	2 (4.6%)	2 (4.4%)	
Easy to stick to my plans and achieve goals			.56^a^
Not true, *n* (%)	1 (2.3%)	1 (2.2%)	
True a few times, *n* (%)	10 (22.7%)	7 (15.2%)	
True more times, *n* (%)	18 (40.9%)	26 (56.5%)	
Exactly true, *n* (%)	13 (29.6%)	11 (23.9%)	
Missing, *n* (%)	2 (4.6%)	1 (2.2%)	
Confident that I can deal with unexpected situations			.21^a^
Not true, *n* (%)	3 (6.8%)	0 (0.0%)	
True a few times, *n* (%)	6 (13.6%)	6 (13.0%)	
True more times, *n* (%)	25 (56.8%)	25 (54.4%)	
Exactly true, *n* (%)	8 (18.2%)	14 (30.4%)	
Missing, *n* (%)	2 (4.6%)	1 (2.2%)	
With my personal resources, I know how to handle situations			.02^a^
Not true, *n* (%)	3 (6.8%)	0 (0.0%)	
True a few times, *n* (%)	7 (15.9%)	1 (2.2%)	
True more times, *n* (%)	19 (43.2%)	27 (58.7%)	
Exactly true, *n* (%)	12 (27.3%)	18 (39.1%)	
Missing, *n* (%)	3 (6.8%)	0 (0.0%)	
I can solve most problems if I do enough for it			.05^a^
Not true, *n* (%)	0 (0.0%)	0 (0.0%)	
True a few times, *n* (%)	2 (4.6%)	2 (4.4%)	
True more times, *n* (%)	25 (56.8%)	16 (34.8%)	
Exactly true, *n* (%)	14 (31.8%)	27 (58.7%)	
Missing, *n* (%)	3 (6.8%)	1 (2.2%)	
I keep calm, because I know I can solve problems			.57^a^
Not true, *n* (%)	0 (0.0%)	0 (0.0%)	
True a few times, *n* (%)	6 (13.6%)	5 (10.9%)	
True more times, *n* (%)	24 (54.6%)	23 (50.0%)	
Exactly true, *n* (%)	12 (27.3%)	18 (39.1%)	
Missing, *n* (%)	2 (4.6%)	0 (0.0%)	
When I find a problem, I usually find more solutions			.05^a^
Not true, *n* (%)	1 (2.3%)	0 (0.0%)	
True a few times, *n* (%)	6 (13.6%)	1 (2.2%)	
True more times, *n* (%)	25 (56.8%)	24 (52.2%)	
Exactly true, *n* (%)	10 (22.7%)	20 (43.5%)	
Missing, *n* (%)	2 (4.6%)	1 (2.2%)	
When I am in trouble, I usually find a way out			.03^a^
Not true, *n* (%)	0 (0.0%)	0 (0.0%)	
True a few times, *n* (%)	3 (6.8%)	0 (0.0%)	
True more times, *n* (%)	27 (61.4%)	22 (47.8%)	
Exactly true, *n* (%)	12 (27.3%)	24 (52.2%)	
Missing, *n* (%)	2 (4.6%)	0 (0.0%)	
No matter what happens, I can deal with it			.07^a^
Not true, *n* (%)	1 (2.3%)	0 (0.0%)	
True a few times, *n* (%)	5 (11.4%)	7 (15.2%)	
True more times, *n* (%)	24 (54.6%)	15 (32.6%)	
Exactly true, *n* (%)	12 (27.3%)	24 (52.2%)	
Missing, *n* (%)	2 (4.6%)	0 (0.0%)	

*N* = 88 (Missing = 2)	Individual information (*n* = 42)	Group information (*n* = 46)	*p*‐value
Self‐efficacy mean sum score			
Sum score (mean, *SD*)	30.9 (5.1)	32.8 (4.3)	.16^a^

^a^ Chi‐squared test.

### 4.5 Resources

For the individual information, the resources in terms of nursing hours are 1 hr per patient, including preparation, education/information and documentation. The use of resources in nursing hours for the group information is calculated at 2.5 hr per week, including preparation, travel time, education/information, documentation and cleaning up the room, regardless of how many patients and family caregivers attend. Thereby, four patients and family caregivers or more had to attend to save costs.

## 5 DISCUSSION

Our goal was to maintain a high level of information obtained by the patients because chemotherapy treatment referral is shown to be the period with most anxiety (Lim et al., 2011). We wanted to enable them to make informed decisions and be in charge of their own lives, even though the change of informational practice would reduce nursing resources. Another aim of the study was to give patients and family caregivers the opportunity to interact and learn from peers; however, we did not collect data on a family level, which could be informative for future studies.

In this prospective comparative study, we included most patients with breast cancer referred for treatment within the 6‐month inclusion period. A minor number of patients were excluded due to mental problems, and only five patients did not want to participate in the study.

The satisfaction with perceived *Knowledge* was high in both groups, but, surprisingly, the patients had an even higher level of knowledge needed than they perceived about breast cancer. However, this is in concordance with other studies as patients with breast cancer in general have high needs for information (Knobf, 2015), which is closely connected to higher levels of education (Fiszer et al., 2014). A systematic review by Fiszer et al. also revealed that informational needs like “being informed about things you can do to get well” are ranked as very important for patients with breast cancer (Fiszer et al., 2014). A few patients in our study, equally in both groups, noted in the comment box in the questionnaire that this specific need—to be informed about things you can do yourself—was not completely fulfilled. According to Blodt et al., information regarding illness experience is closely associated with gaining control in a seemingly uncontrollable situation to avoid the disease taking over. However, there is always a fine line between information seeking and the risk of becoming overwhelmed by information (Blodt et al., 2018; Ormel et al., 2020). The challenge for the nurses is to fit the information to the individual patient's need. Thus, with the novel practice of group information, it may be even more demanding for the nurses to assess the individual needs for each patient and navigate what information to give in the group and what information to give in person.

In relation to ***Support***, the patients found the physical needs best supported by the healthcare professionals, which may also be the most concrete problems to describe and handle.

A relatively large number did not find it relevant to seek support for family problems or sexual problems, although sexual problems are known to be closely linked to breast cancer (Carroll et al., 2016). However, this questionnaire measured only a snapshot early in a long treatment trajectory; further, the sensitive nature of the questions may be a taboo in Denmark and require a more trusting relationship with the nurse.

We had hypothesized peer support to be better in the group information, but it only improved a few per cent. However, compared with Bjorneklett et al. who had a 1‐week support group (Bjorneklett et al., 2012), our study participants spent only a short time together. In future studies, peer support could be increased and made even more systematic by booking the patients for chemotherapy in the same treatment rooms.

The *Ability to act* in everyday life assessed by the GSE self‐efficacy scale was in general with high levels in both groups, although we saw a trend towards an optimized self‐efficacy outcome in the patients receiving group information. Rottmann et al. found higher self‐efficacy in breast cancer as a significant predictor of an active adjustment style and emotional well‐being after 12 months (Rottmann et al., 2010), that is this measure is important to monitor in breast cancer treatment. Compared with Rottmann et al., who found a mean sum score at baseline 27.4 increasing to 27.9 at 1‐month follow‐up, our patients had a high self‐efficacy sum score in the individual information group at 30.9 and the group information at 32.8. However, this may be due to differences in the two study populations. Circumstances such as age, comorbidity, prognosis, social status and mental state when answering the questions may affect the outcome. Higher baseline of self‐efficacy may indicate that the study population in this study was more resourceful and therefore had a high ability to act accordingly. The greater increase in this study in comparison with the study by Rottmann et al. may be due to length in follow‐up time or differences in the way the questions were executed.

In summary, even though the intervention did not prove to be significantly better in all aspects, it is important to note that nor was the intervention worse. This could indicate that group information is a good option when aiming to optimize the use of scarce resources in the healthcare sector without compromising the quality of the health care provided. The resources saved when giving group information can be allocated to other areas and thereby generate even greater overall benefits. The demographic changes in the population, combined with the increasing prevalence of cancer and prolonged survival (Jørgensen, 2015; Danckert et al., 2018), require solutions such as group information to help ensure high quality in health care in the future.

The resources used in nursing hours in the individual information group versus the group information ended up being neutral in our department on a weekly basis because there were only two‐three new patients with breast cancer per week. Indeed, there is a possibility of cost savings as it is manageable to have more patients and family caregivers in the group information, which is also shown in van Ooij Oostrom et al. (2010), where they only executed the group information every second week. However, Danish Health Authority regulations are very strict about initiating early treatment for cancer; therefore, we decided to offer group information each week.

### 5.1 Strength and limitations

To our knowledge, this is the first study investigating level of knowledge and support after group information for patients with breast cancer, which could inform future informational practice. Very few patients declined to participate although the patients were in a very stressful situation and this is a huge strength in the study. However, a limitation to consider was the relatively large number of women *N* = 23 who were omitted from the study by the pressure of busyness or forgetfulness by clinical staff. While regrettable, this is a known issue when executing research in real‐time clinical practice (Cox & McGarry, 2003). However, research led by nurses is increasing in cancer care (Charalambous et al., 2018), so it is important to consider the inclusion of patients in research as a core area in nursing care.

Future experimental information studies could benefit from the randomized controlled design. However, the strength of the simple comparative design we used is to suggest that intervention has an impact on the outcome, but the design has no control over other things changing during the same period (Thiese, 2014), and unfortunately, we did not collect baseline data. However, we believe the design to be relatively strong in this study, as the period for enrolment was relatively narrow, and the treatment programme was stable within the period. Particularly important is that the two groups were comparable in age and educational level, and both groups had the same access to other sources of information. Further, the short time point for evaluation reduced the risk of recall bias.

Another strength was the use of the validated GSE self‐efficacy scale, which covers a broad range of the sense of personal competence needed to deal effectively with stressful situations and which allows us to compare with other studies. Further, the GSE self‐efficacy has been used several times among breast cancer patients in Denmark (Debess et al., 2009; Rottmann et al., 2010). However, a drawback was that at the time of study planning, we could not find validated questionnaires fitting the wanted questions on *Knowledge* of breast cancer and perceived *Support*, but the reports from the few patients who piloted the material and from included patients reveal that the questions developed were easy to answer.

A minor limitation to this study is the relatively small sample size (i.e. 44 in the individual information and 46 in the group information), although the groups are bigger than suggested by Collins et al. (2007), which recommends at least 21 persons per group for experimental studies. Also, the strength of the study would have increased if it were possible for the participants to function as their own control (crossover design) and this would also have eliminated the possibility of confounding and between‐group differences (Mills et al., 2009). However, this is not always possible in real life; further, by not using a crossover design, the chance of carryover effects is eliminated.

The group information was non‐inferior compared with individual information, but some self‐efficacy outcomes were increased in the group information. As the goal was to maintain a high level of quality in the perceived information and the patients were very satisfied in both groups, we conclude that group information was cost‐effective and safe to perform.

## CONFLICT OF INTEREST

The authors declare no conflicts of interest.

## AUTHOR CONTRIBUTIONS

Dieperink KB conceptualized, formally analysed, investigated the study, involved in methodology and project administration and wrote original draft. Ellegaard E and Astrup AL conceptualized and involved in data inclusion and review and editing. Hasse HT and Elnegaard CM formally analysed, investigated and involved in methodology and original draft preparation. Jensen JD involved in conceptualization, methodology, project administration, supervision, validation and writing—review and editing.
